# Genetic landscape and phenotypic correlations of lissencephaly: prenatal and postnatal insights

**DOI:** 10.1093/braincomms/fcag069

**Published:** 2026-03-06

**Authors:** Ruibin Huang, Fang Fu, Na Zhang, Hang Zhou, Shanshan Mei, Jin Han, Qiong Deng, Hongsheng Liu, Yongling Zhang, Qiuxia Yu, Min Pan, Fucheng Li, Jianqin Lu, Chunling Ma, Fei Guo, Huanyi Chen, Liyuan Liu, Xinyi Zhao, Xinyue Tan, Dongzhi Li, Ru Li, Can Liao

**Affiliations:** Prenatal Diagnostic Center, Guangzhou Women and Children’s Medical Center, Guangzhou Medical University, Guangzhou, Guangdong 510623, China; Prenatal Diagnostic Center, Guangzhou Women and Children’s Medical Center, Guangzhou Medical University, Guangzhou, Guangdong 510623, China; Prenatal Diagnostic Center, Guangzhou Women and Children’s Medical Center, Guangzhou Medical University, Guangzhou, Guangdong 510623, China; Department of Obstetrics and Gynecology, Affiliated Hospital of Guangdong Medical University, Zhanjiang, Guangdong 524002, China; Prenatal Diagnostic Center, Guangzhou Women and Children’s Medical Center, Guangzhou Medical University, Guangzhou, Guangdong 510623, China; Department of Obstetrics and Gynecology, Guangzhou Women and Children’s Medical Center, Guangzhou Medical University, Guangzhou, Guangdong 510623, China; Prenatal Diagnostic Center, Guangzhou Women and Children’s Medical Center, Guangzhou Medical University, Guangzhou, Guangdong 510623, China; Prenatal Diagnostic Center, Guangzhou Women and Children’s Medical Center, Guangzhou Medical University, Guangzhou, Guangdong 510623, China; Department of Radiology, Guangzhou Women and Children’s Medical Center, Guangzhou Medical University, Guangzhou, Guangdong 510623, China; Prenatal Diagnostic Center, Guangzhou Women and Children’s Medical Center, Guangzhou Medical University, Guangzhou, Guangdong 510623, China; Prenatal Diagnostic Center, Guangzhou Women and Children’s Medical Center, Guangzhou Medical University, Guangzhou, Guangdong 510623, China; Prenatal Diagnostic Center, Guangzhou Women and Children’s Medical Center, Guangzhou Medical University, Guangzhou, Guangdong 510623, China; Prenatal Diagnostic Center, Guangzhou Women and Children’s Medical Center, Guangzhou Medical University, Guangzhou, Guangdong 510623, China; Prenatal Diagnostic Center, Guangzhou Women and Children’s Medical Center, Guangzhou Medical University, Guangzhou, Guangdong 510623, China; Prenatal Diagnostic Center, Guangzhou Women and Children’s Medical Center, Guangzhou Medical University, Guangzhou, Guangdong 510623, China; The First Clinical Medical College, Southern Medical University, Guangzhou, Guangdong 510515, China; Prenatal Diagnostic Center, Guangzhou Women and Children’s Medical Center, Guangzhou Medical University, Guangzhou, Guangdong 510623, China; Prenatal Diagnostic Center, Guangzhou Women and Children’s Medical Center, Guangzhou Medical University, Guangzhou, Guangdong 510623, China; Prenatal Diagnostic Center, Guangzhou Women and Children’s Medical Center, Guangzhou Medical University, Guangzhou, Guangdong 510623, China; The First Clinical Medical College, Southern Medical University, Guangzhou, Guangdong 510515, China; Prenatal Diagnostic Center, Guangzhou Women and Children’s Medical Center, Guangzhou Medical University, Guangzhou, Guangdong 510623, China; Prenatal Diagnostic Center, Guangzhou Women and Children’s Medical Center, Guangzhou Medical University, Guangzhou, Guangdong 510623, China; Prenatal Diagnostic Center, Guangzhou Women and Children’s Medical Center, Guangzhou Medical University, Guangzhou, Guangdong 510623, China; Prenatal Diagnostic Center, Guangzhou Women and Children’s Medical Center, Guangzhou Medical University, Guangzhou, Guangdong 510623, China; Prenatal Diagnostic Center, Guangzhou Women and Children’s Medical Center, Guangzhou Medical University, Guangzhou, Guangdong 510623, China

**Keywords:** agyria, pachygyria, subcortical band heterotopia, whole exome sequencing, prenatal diagnosis

## Abstract

Lissencephaly (LIS) is a spectrum of cortical malformations including agyria, pachygyria and subcortical band heterotopia, which arises from aberrant neuronal migration and is associated with severe neurodevelopmental impairments. Despite advancements in prenatal imaging, diagnosing LIS remains challenging. Genetic factors play a crucial role in LIS, involving multiple genes and signalling pathways, yet research on prenatal diagnosis and the genetic basis is still limited. This study aimed to assess the diagnostic yield of whole exome sequencing (WES) in LIS and to examine genotype−phenotype correlations, addressing the challenge of ‘phenotype lag’ in prenatal LIS diagnosis. This study included 20 fetuses with LIS suggested by prenatal imaging and 20 children with LIS diagnosed after birth; all cases were diagnosed by magnetic resonance imaging and underwent genetic testing. In addition, a literature review was conducted and 80 studies were included, of which 1 was used to compare detection efficacy and 79 studies totalling 210 cases were used to assess genotype−phenotype correlation. In the prenatal cohort, 85.0% (17/20) of cases exhibited concurrent anomalies, predominantly ventriculomegaly (50.0%) and microcephaly (25.0%). In the postnatal cohort, the most common phenotypes were epilepsy (80.0%, 16/20) and global developmental delay (65.0%, 13/20), with half of the cases (10/20) showing no abnormalities in the prenatal period. The diagnostic yields were 55.0% (11/20) and 65.0% (13/20), respectively, with *PAFAH1B1* point mutations or 17p13.3 microdeletions being the predominant genetic variant in both cohorts, accounting for 31.3% (prenatal) and 25.5% (postnatal) of cases, respectively. *DARS2* and *NPRL3* were reported to be associated with LIS for the first time in this study. Literature synthesis revealed an overall diagnostic yield of 79.04%, dominated by *PAFAH1B1* (26.3%), *DYNC1H1* (11.9%), and *DCX* (10.2%). By reviewing the prenatal images, up to 48.05% (74/154) of the cases had no specific findings in the prenatal period, and the most common presentations were ventriculomegaly/hydrocephalus (52.63%) and head circumference anomalies (29.82%). This study highlights the significant genetic heterogeneity, phenotypic complexity and diagnostic challenges of LIS by integrating data from our cohort and the published literature. We developed a comprehensive genetic aetiology classification framework for LIS and identified novel associations with non-canonical genes such as *NPRL3* and *DARS2*. With a high molecular diagnostic yield of 79.04%, we recommend WES as the first-line genetic test. Furthermore, the establishment of an integrated prenatal imaging-molecular diagnostic system, along with a postnatal multidisciplinary model, is crucial for improving prognosis assessment, clinical decision-making and genetic counselling.

## Teaser

Huang *et al*. report that by integrating prenatal and postnatal data, they identified significant genetic heterogeneity, phenotypic complexity and challenges in prenatal diagnosis for lissencephaly. They recommend whole exome sequencing as the first-line diagnostic method to improve genetic counselling and prognosis assessment.

## Graphical Abstract

**Graphical Abstract:**
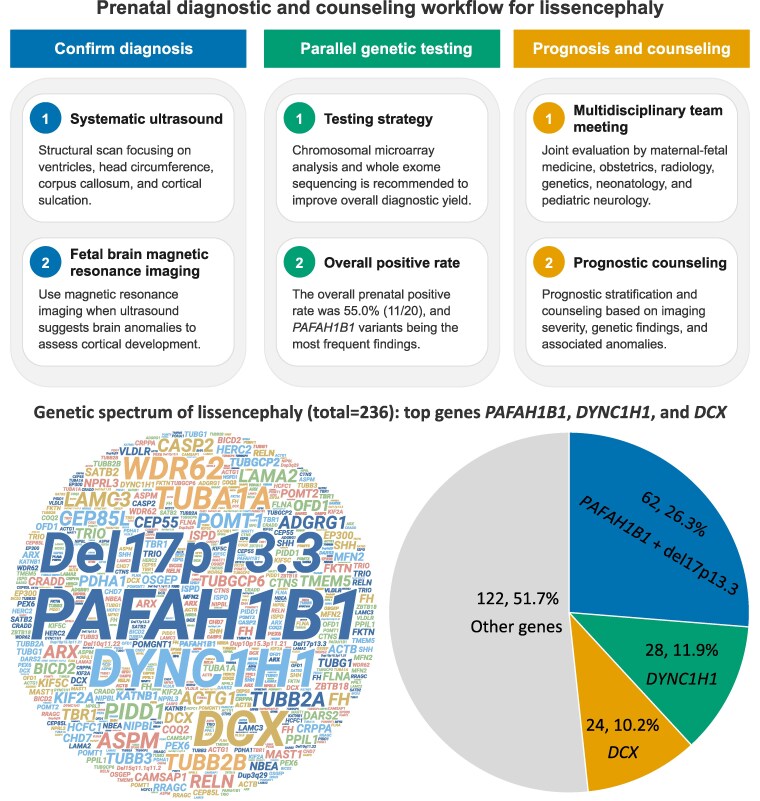

## Introduction

Lissencephaly (LIS) comprises a spectrum of rare structural brain disorders, including agyria, pachygyria and subcortical band heterotopia (SBH), and is characterized by absent or simplified cerebral gyri and thickening of the cortical grey matter.^1,2^ The incidence of LIS in neonates is approximately 1–4 per 100 000,^3^ but there are currently no data on the incidence during the fetal period. LIS pathogenesis primarily stems from aberrant neuronal migration during early corticogenesis, and is affected by multiple factors such as genetics, epigenetics and the environment,^4^ with genetic factors involving multiple genes in multiple signalling pathways, including *PAFAH1B1*, *DCX*, *TUBA1A*, etc^5–7^. The major clinical manifestations include refractory epilepsy and intellectual disability.^8-10^

Prenatal ultrasound diagnosis of LIS presents certain challenges, primarily relying on the assessment of brain surface smoothness, sulcal development and ventricular morphology.^11^ Prenatal magnetic resonance imaging (MRI) is an important adjunctive diagnostic tool, capable of distinguishing between pachygyria and agyria, which represent different severities of malformations and can coexist.^12-15^ SBH, characterized by subcortical grey matter heterotopia with preserved cortical architecture, is the mildest variant of LIS and is difficult to detect prenatally.^16,17^ Despite advancements in prenatal imaging, missed or misdiagnosed of LIS remains possible, necessitating additional imaging features for diagnostic assistance. Furthermore, although chromosomal microarray analysis (CMA) and whole exome sequencing (WES) have been widely used to detect the genetic burden of fetal structural abnormalities, studies on LIS are still limited to case reports with limited statistical power,^18-21^ with no systematic cohort studies available, and a lack of comprehensive integration of pathogenic genes and variants from the published literature.

This study included 20 prenatally suggested LIS cases and 20 postnatally diagnosed LIS cases, and also performed a systematic review of the published literature. By integrating prenatal−postnatal phenotypic data and molecular profiles, we assessed the detection efficacy of WES and clarified the genotype−phenotype correlation. Furthermore, by reviewing prenatal imaging features of postnatal cases, we addressed the ‘phenotype lag’ issue in prenatal diagnosis of LIS, shifting from passive identification to proactive prediction, thus optimizing the prenatal diagnostic pathway and genetic counselling.

## Materials and methods

### Study cohort

This retrospective study analysed 40 LIS cases referred to Guangzhou Women’s and Children’s Medical Center, Guangzhou Medical University (GWCMC) from January 2019 to December 2024. The study received ethical approval from GWCMC’s Institutional Review Board (Registration number: No.2021-356B01), with written informed consent obtained from all participants.

**Figure 1:**
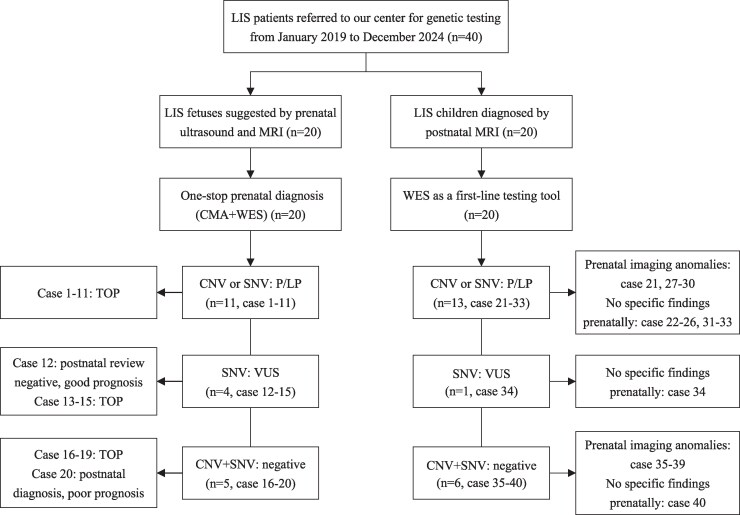
**Diagnostic flowchart of the study and results overview.** LIS, lissencephaly; MRI, magnetic resonance imaging; QF-PCR, quantitative fluorescent polymerase chain reaction; CMA, chromosomal microarray analysis; WES, whole exome sequencing; CNV, copy number variant; P/LP, pathogenic/likely pathogenic; VUS, variants of unknown significance; SNV, single nucleotide variant; TOP, termination of pregnancy.

The study population included two cohorts: (i) 20 fetuses meeting prenatal imaging criteria for LIS, and (ii) 20 paediatric cases with postnatal LIS diagnoses, all confirmed by cranial MRI. A diagnostic flow chart for this study is shown in Fig. 1. For prenatal cases, prenatal imaging and genetic testing results were systematically documented, as well as pregnancy outcomes and prognosis (where available); for postnatal cases, a comprehensive clinical phenotype was collected, including neuroimaging, developmental assessment, longitudinal follow-up and genetic testing results.

### One-stop prenatal diagnosis

For prenatal cases, invasive diagnostic procedures were performed following standardized protocols and after written informed consent was abtained. Fetal samples were collected from amniocytes or cord blood, and parental samples from peripheral blood. Quality control measures included quantitative fluorescent polymerase chain reaction (QF-PCR) to exclude maternal cell contamination and screen for common aneuploidies. Subsequently, a one-stop diagnostic approach was employed, combining CMA and WES to simultaneously detect chromosomal abnormalities and monogenic disorders. Laboratory turnaround time was 14–21 days, ensuring timely pregnancy management decisions. Postnatal cases underwent WES as first-tier testing following international guidelines.^22^ Detailed protocols are provided in Supplementary File 1.

### Literature search strategy

A systematic search was conducted in PubMed, Scopus and Web of Science up to 7 April 2025, to identify studies on LIS, CMA or next-generation sequencing (NGS). Supplementary Fig. 1 depicts the selection workflow, with full methodological details available in Supplementary File 2.

### Statistical analysis

Statistical analyses were primarily descriptive. Continuous variables (e.g. maternal age and gestational week) were summarized as median (range), and categorical variables were summarized as counts and percentages. All percentages were calculated as n/N, with the denominator (N) explicitly stated in the main text or figure legends. The diagnostic yield of CMA, WES and the combined testing strategy was defined as the proportion of tested individuals in whom pathogenic/likely pathogenic (P/LP) variants were identified. For the integrated cohort-literature analyses, gene- and category-level frequencies were primarily calculated using the variant as the experimental unit (with the total number of P/LP variants as the denominator; see figure legends). Overlap analyses in Venn diagrams were performed using the gene as the experimental unit, defined as genes harbouring at least one P/LP variant. Given the retrospective design and limited sample size, no formal hypothesis testing was performed.

## Results

### Prenatal cohort

The prenatal cohort consisted of 20 LIS cases, including 12 females (60.0%) and 8 males (40.0%). The median maternal age was 29.5 years (range: 20–37 years), and the median gestational age at LIS detection was 29.0 gestational weeks (GW) (range: 24–35 GW). Five cases (25.0%, cases 1, 10, 11, 13, and 18) were detected in the second trimester, while the remaining 15 cases (75.0%) were detected in the third trimester. Based on imaging findings, the cases were classified into isolated LIS and non-isolated LIS. Three cases (15.0%) were isolated LIS, while 17 cases (85.0%) presented with concurrent anomalies. Additionally, 70.0% (14/20) presented with other cranial abnormalities, primarily ventriculomegaly/hydrocephalus (50.0%, 10/20) and microcephaly (25.0%, 5/20).

CMA identified six (30.0%, 6/20) pathogenic copy number variants (pCNVs), with five cases carried 17p13.3 microdeletions and one case with a 1q42.2q43 deletion (Table 1). All above cases were confirmed as *de novo* variants through WES. Additionally, Case 7 showed a 10p12.31 microduplication, classified as variants of unknown significance (VUS). Familial analysis indicated its inheritance from the phenotypically normal father. WES identified P/LP single nucleotide variants (p/lpSNVs) in four cases (4/20, 20.0%), all of which were *de novo* variants (Supplementary Table 1). Additionally, Case 11 carried a compound heterozygous variant in the *DARS2* gene, classified as LP and VUS; Cases 12–15 carried VUS inherited from unaffected parents. Overall, the diagnostic yield from CMA and WES was 55.0% (11/20).

**Table 1:** Prenatal diagnosis of LIS fetuses: clinical features, CNV profiling and pregnancy outcomes

Case	MA (y)	GW (wk)	Ultrasound findings	CMA findings	Size (Mb)	Interpretation	Inheritance	Outcomes
1	37	25.4	Agyria	arr[GRCh37]17p13.3p13.1(525–6 624 339) × 1	6.62	Pathogenic	De novo	TOP
2	28	29.7	Pachygyria; Ventriculomegaly (R); Microcephaly	arr[GRCh37]17p13.3p13.2(526–3 522 432) × 1	3.52	Pathogenic	De novo	TOP
3	28	28.9	Pachygyria; Ventriculomegaly (B)	arr[GRCh37]17p13.3(1 903 484–3 247 426) × 1	1.34	Pathogenic	De novo	TOP
4	28	27.9	Agyria; Choroid plexus cyst; Polyhydramnios	arr[GRCh37]17p13.3(1 594 925–3 063 414) × 1	1.47	Pathogenic	De novo	TOP
5	35	31.0	Agyria; Microcephaly; ARSA	arr[GRCh37]17p13.3p13.2(2 139 710–3 337 135) × 1	1.20	Pathogenic	De novo	TOP
6	27	29.7	Pachygyria; Pericardial effusion	arr[GRCh37]1q42.2q43(231 096 955–242 687 578) × 1	11.59	Pathogenic	De novo	TOP
7	26	34.4	Pachygyria; Ventriculomegaly (B); Polyhydramnios	arr[GRCh37]10p12.31(19 443 445–20 475 210) × 3	1.03	VUS	Paternal	TOP

LIS, Lissencephaly; CNV, copy number variant; MA, maternal age; GW, gestational weeks; CMA, chromosomal microarray analysis; TOP, termination of pregnancy; R, right; B, bilateral; ARSA, aberrant right subclavian artery; VUS, variants of unknown significance.

Following genetic counselling, 90% (18/20) of families chose pregnancy termination. On one hand, most postnatal LIS cases lead to abnormalities such as epilepsy and intellectual disability (ID), which cannot be predicted prenatally. On the other hand, most LIS fetuses present with additional anomalies, indicating a higher risk postnatally. Cases 12 and 20 chose to continue their pregnancies. Case 12 had a favourable outcome with normal growth and no LIS signs at 2 months, while case 20 developed intracranial haemorrhage, porencephaly and other abnormalities, leading to cerebral palsy and intellectual disabilities with a poor prognosis.

### Postnatal cohort

The postnatal cohort included 20 LIS cases, predominantly male (11 males, 55.0%; 9 females, 45.0%), diagnosed at a median age of 3 months (range: 0–12 months). Additional intracranial abnormalities were observed in 40.0% (8/20) of cases, including cerebellar hypoplasia (2), corpus callosum agenesis (2), polymicrogyria (2), white matter injury (1) and complex malformations (1). The main clinical manifestations were epilepsy (80.0%, 16/20) and global developmental delay (GDD) (65.0%, 13/20). Retrospective prenatal analysis revealed 50.0% (10/20) with normal imaging, 20.0% (4/20) with ventriculomegaly and 15.0% (3/20) with fetal growth restriction (FGR). Other findings included decreased fetal movement in Case 29, left foot eversion in Case 36 and oligohydramnios in Case 37. Additionally, three cases (28–30) experienced intrauterine distress, and Cases 34 and 37 had placental abruption and fever during delivery, respectively.

WES identified P/LP variants in 65% (13/20) of cases. Seven cases had 17p13.3 microdeletions or *PAFAH1B1* gene point mutations, and three cases exhibited variants linked to Lissencephaly types 2/3 or complex cortical dysplasia (Supplementary Table 2). Case 23 had a *de novo* 3q29 microduplication, classified as likely pathogenic. Case 30 carried a homozygous c.740G > A (p.Arg247Gln) variant in the *OSGEP* gene, associated with Galloway−Mowat syndrome 3. Case 29 harboured an *NPRL3* gene c.318 + 1G > T variant, classified as pathogenic, linked to familial focal epilepsy with variable foci-3 (FFEVF3). Case 34 had an *NPRL2* gene c.34_36del (p.Phe12del) variant, classified as VUS, associated with familial focal epilepsy with variable foci-2 (FFEVF2).

### Literature review and summary

#### Study selection

A systematic review of 1125 articles across PubMed, Scopus and Web of Science identified 80 relevant studies, after applying inclusion/exclusion criteria and excluding duplicates. Full details of the selection process are provided in Supplementary Fig. 1 and Supplementary File 3.

#### Study characteristics

The review included studies from 28 countries across six continents. Of 80 studies, 7 were cohort studies,^18-21,23-25^ 5 were case series and 68 were case reports, with 8 prenatal and 72 postnatal investigations. Notably, the study by Di Donato *et al*.^19^ was the first large multi-centre cohort study. To avoid bias from its sample size, its data were excluded from statistical analysis of pathogenic genes and variants, and used only for comparison of detection efficiency. The remaining 79 studies involved 210 LIS patients. Detailed study characteristics are provided in Supplementary File 3.

#### Overall detection results

Seven cohort studies with 903 LIS patients showed a diagnostic rate of 79.04% (range: 58.33–90.91%), with 717 confirmed diagnoses. Wang *et al*.^24^ used CMA and WES for 16 LIS fetuses with a detection rate of 75.00% (12/16). Postnatal cohorts with targeted gene panels showed a detection rate of 79.93% (range: 60.88–80.76%).^18,19,23^ Two studies using WES as first-line testing achieved a 75.00% (range: 64.70–90.91%) detection rate^21,25^ and one study using genomic sequencing post-WES had a 58.33% detection rate.^20^

**Figure 2:**
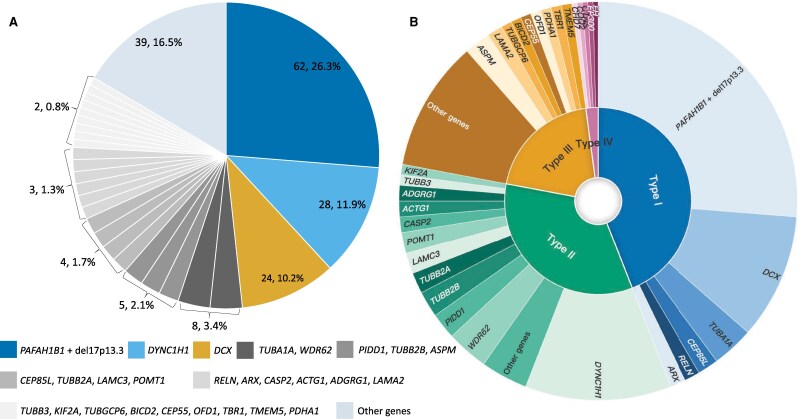
**Distribution and classification of LIS-related genes.** (**A**) Distribution of pathogenic/likely pathogenic (P/LP) findings across LIS-associated genes. Data were compiled from 24 molecularly diagnosed cases in our cohort and 210 cases identified through the literature review, yielding a total of *N* = 236 P/LP variants (experimental unit = variant; two cases contributed two independent P/LP variants). Numbers and percentages shown in the pie chart indicate the variant count and proportion for each gene (n/N). The three most frequently affected genes were *PAFAH1B1* (including point mutations and 17p13.3 microdeletions) (62/236, 26.3%), *DYNC1H1* (28/236, 11.9%) and *DCX* (24/236, 10.2%). (**B**) Classification of the same *N* = 236 P/LP variants into four categories based on their relevance to LIS: Type **I**, major-effect genes primarily associated with LIS (104/236, 44.1%); Type II, genes related to cortical malformations (80/236, 33.9%); Type III, genes associated with neurodevelopmental disorders (47/236, 19.9%); and Type IV, genes mainly linked to multisystem abnormalities (5/236, 2.1%). Percentages indicate the proportion of variants in each category (n/N; experimental unit = variant).

Our cohort analysis (24 confirmed cases and 210 reviewed) identified 236 pathogenic variants, with two cases (Cases 163 and 181) showing dual positive diagnoses. As illustrated in Fig. 2A, key variants included *PAFAH1B1* point mutations or 17p13.3 microdeletions (26.3%, 62/236), *DYNC1H1* (11.9%, 28/236) and *DCX* (10.2%, 24/236), collectively representing 48.3% of positive variants. Additionally, eight cases carried mutations in *TUBA1A* and *WDR62* (3.4%).

We categorized the variants into four types based on pathogenic relevance to LIS (Fig. 2B): Type I (Major-effect genes specific to LIS): *PAFAH1B1* point mutations or 17p13.3 microdeletions (*n* = 62), *DCX* (*n* = 24), *TUBA1A* (*n* = 8), *CEP85L* (*n* = 4), *RELN* (*n* = 3) and *ARX* (*n* = 3), collectively accounting for 44.1% (104/236) of the detected variants. Type II (Cortical malformation-related genes): *DYNC1H1* (*n* = 28), *WDR62* (*n* = 8), *PIDD1* (*n* = 5), *TUBB2B* (*n* = 5) and *TUBB2A* (*n* = 4), accounting for 33.9% (80/236) of the variants detected. Type III (neurodevelopmental disorder-related genes): *ASPM* (*n* = 5), *LAMA2* (*n* = 3), *TUBGCP6* (*n* = 2), *BICD2* (*n* = 2), *CEP55* (*n* = 2), *OFD1* (*n* = 2), *PDHA1* (*n* = 2) and *TBR1* (*n* = 2), accounting for 19.9% (47/236) of the variants. Type IV (multi-system abnormality-related genes): *CHD7* (*n* = 1), *COQ2* (*n* = 1), *CTNS* (*n* = 1), *EP300* (*n* = 1) and *FH* (del1q42.2q43) (n = 1), accounting for 2.1% (5/236) of the total variants detected. It is noteworthy that gene mutations in *ASPM*, *TUBGCP6*, *BICD2*, *OFD1*, *PDHA1* and *TBR1* in Type III were detected in at least two independent studies. Additionally, the potential pathogenicity of the genes in Type IV, including *CHD7*, *COQ2*, *CTNS*, *EP300* and *FH*, suggests that these 11 genes may be potential candidate pathogenic genes for LIS.

**Figure 3:**
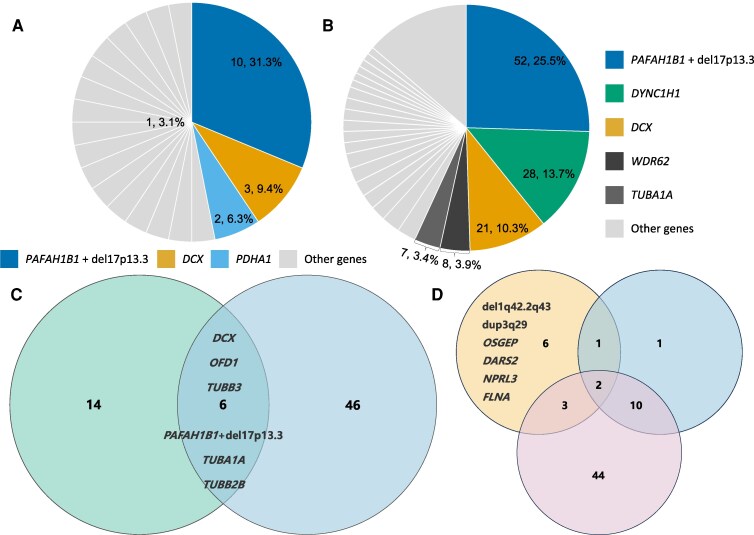
**Genetic spectrum of prenatal versus postnatal lissencephaly cases.** (**A**) Distribution of genes harbouring P/LP variants in the prenatal group (*N* = 32 variants). (**B**) Distribution of genes harbouring P/LP variants in the postnatal group (*N* = 204 variants). Numbers and percentages represent the variant count and proportion for each gene (n/N). (**C**) Venn diagram showing the overlap of genes with P/LP variants between prenatal and postnatal groups (experimental unit = gene): *N* = 20 genes in the prenatal group (left circle), *N* = 52 genes in the postnatal group (right circle), with six shared genes. (**D**) Venn diagram comparing LIS-associated genes reported in the current study (upper-left circle), Di Donato *et al*. (upper-right circle), and other published studies (lower circle) (experimental unit = gene; the total number of genes in each dataset is indicated in the diagram).

Genetic distributions between prenatal and postnatal cases showed that *PAFAH1B1*, *DCX* and microtubule protein genes are the core genetic causes of LIS, with *PAFAH1B1* point mutations or 17p13.3 microdeletions being the predominant variant in both groups (31.3% in prenatal and 25.5% in postnatal cases) (Figs 3A and B and 3C). *DCX* variants maintained comparable frequencies in both groups (9.4% and 10.3%, respectively). *DYNC1H1* variants were more common in postnatal cases (13.7%), suggesting potential under-detection prenatally due to milder phenotypes or later onset of symptoms.

**Figure 4:**
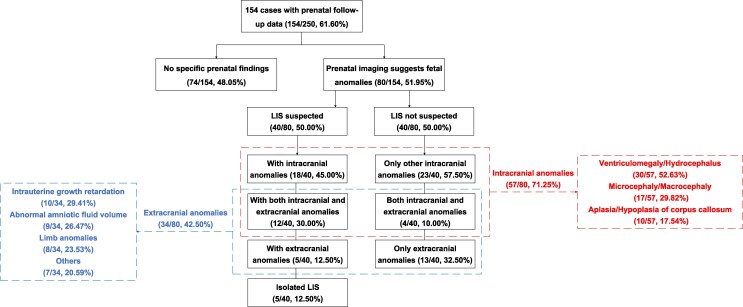
**Comprehensive overview of prenatal and postnatal phenotypic characteristics of lissencephaly**. LIS, lissencephaly.

The phenotypic distribution of LIS cases is summarized in Fig. 4. Among the 250 cases included, 154 (61.6%) had prenatal follow-up information. Of these, 74.03% (114/154) of LIS cases had no LIS detected prenatally, nearly half (48.05%, 74/154) showed no abnormalities during prenatal screening and 51.95% (80/154) had prenatal anomalies. Intracranial comorbidities in 57 cases (71.25%) mainly included ventriculomegaly/hydrocephalus (52.63%), microcephaly/macrocephaly (29.82%) and corpus callosum hypoplasia (17.54%). Extracranial anomalies (42.5%) were often intrauterine growth restriction (29.41%), abnormal amniotic fluid volume (26.47%) and limb malformations (23.53%). Postnatal phenotypic data were available for 178 cases (71.2%), with one case (0.56%) reclassified as non-LIS. Neurological abnormalities dominated (94.94%, 169/178), with 71.91% (128/178) having only neurological involvement, 23.03% (41/178) multi-system involvement and 4.49% (8/178) having no neurological symptoms (all deceased in infancy). Epilepsy was present in 76.4% (136/178), followed by GDD (53.37%, 95/178) and ID (35.39%, 63/178). Non-neurological anomalies included craniofacial dysmorphism (42.85%, 21/49), skeletal defects (26.53%, 13/49) and genitourinary abnormalities (16.33%, 8/49).

## Discussion

This study systematically characterizes the prenatal imaging features, genetic profiles and postnatal phenotypic correlations of LIS. In the prenatal cohort, 85% of LIS cases had other structural abnormalities, with ventriculomegaly and microcephaly being the most common. Prenatal diagnoses were primarily linked to 17p13.3 microdeletions and pathogenic variants in LIS-associated genes such as *PAFAH1B1*, yielding a 55.0% genetic detection rate. Postnatally, a broader genetic spectrum emerged, with epilepsy and GDD being prominent phenotypes. Literature review further underscored the marked genetic heterogeneity and phenotypic complexity of LIS.

LIS is commonly associated with canonical genes like *PAFAH1B1*, *TUBA1A* and *DCX* (Figs 3C), but our study highlights its molecular heterogeneity. WES was pivotal in identifying both known variants and rare *de novo* mutations. Comparative analysis of our cohort with 79 published studies and panel sequencing-based research by Di Donato *et al*. (Figure 3D), identified six variants exclusive to our study. For instance, Case 6, with prenatal imaging suggesting pachygyria and pericardial effusion, showed a *de novo* deletion at 1q42.2q43, overlapping genes linked to growth retardation and neurodevelopmental delay. Case 23, with normal prenatal imaging, was diagnosed with GDD postnatally after MRI identified agyria and cerebellar hypoplasia. WES revealed a *de novo* 3q29 duplication. Case 8, with isolated prenatal agyria, had a pathogenic *de novo FLNA* variant (c.2853T > G; p.Tyr951Ter), highlighting the limitations of prenatal imaging in detecting specific cortical malformations due to developmental immaturity or technical constraints. Case 30, diagnosed prenatally with FGR and oligohydramnios, later showed pachygyria and epilepsy on postnatal MRI. WES identified a homozygous pathogenic *OSGEP* variant (c.740G > A; p.Arg247Gln), linked to Galloway−Mowatt syndrome (GAMOS). Although GAMOS is associated with nephropathy and microcephaly, only one prior study has suggested a connection between *OSGEP* mutations and LIS.^26^ This case further supports the hypothesis that *OSGEP* may contribute to LIS, underscoring the importance of long-term monitoring for neurological manifestations in GAMOS patients.

Our study identifies the *NPRL3* and *DARS2* genes as novel associations with LIS. Case 29, which initially showing no abnormalities on prenatal imaging, developed seizures postnatally, with MRI revealing pachygyria and white matter injury. WES identified a pathogenic *de novo* c.318 + 1G > T variant in *NPRL3*, part of the GATOR1 complex, which regulates neuronal migration and cortical lamination by inhibiting mTORC1 signalling.^27^ The c.318 + 1G > T mutation likely causes GATOR1 dysfunction and abnormal mTORC1 activation, a mechanism confirmed in animal models.^28,29^  *NPRL3* mutations are typically associated with FFEVF3, and while imaging may show focal cortical dysplasia or no apparent abnormalities, its direct connection to LIS remains unclear.^8,30-34^ Therefore, this study expands the phenotypic spectrum of the NPRL3 gene, but further case reports are needed to support its association with LIS. Case 11 presented multiple malformations, including agyria, microcephaly and others. WES revealed compound heterozygous variants (c.868C > T [p.Gln290Ter] and c.1862T > C [p.Val621Ala]) in the *DARS2* gene, classified as LP and VUS. *DARS2* is crucial for mitochondrial function,^35^ and its deficiency impairs mitochondrial respiratory chain complex, impairing energy metabolism and organ development.^36^  *DARS2* mutations are typically associated with LBSL, a progressive neurodegenerative disorder affecting the brainstem and spinal cord. However, the phenotype in Case 11 emerged during embryonic development, suggesting that the severity and combination of genetic variants influence clinical manifestation timing. *Dars2* knockout in mice results in Purkinje cell loss and mitochondrial defects, underscoring its essential role in CNS development.^37^ The phenotypic spectrum of *DARS2* mutations is broad, ranging from no apparent phenotype to severe neurological dysfunction, depending on the specific variant and its context. The compound heterozygous variants in this case preserved partial enzymatic function, allowing embryonic survival but not normal organ development.^38,39^ Although these studies suggest a potential link between *DARS2* and the central nervous system, there is unfortunately a lack of further functional studies or additional prenatal case reports to confirm the pathogenicity of this variant. Therefore, while this case is considered a potential diagnostic case, further research is needed to confirm its association with the agyria phenotype.

Prenatal diagnosis of LIS is challenging, with 74.03% of LIS cases undetected prenatally, and 48.05% showing no abnormalities on imaging (Fig. 4). When other fetal anomalies are present, a comprehensive ultrasound should be performed to assess neurological involvement. If neurological signs are detected, particularly ventriculomegaly, abnormal head circumference or corpus callosum abnormalities, a targeted reassessment of the fetal brain is recommended after 28 GW, combining high-resolution ultrasound and MRI to evaluate cortical development. For example, Case 3, with mild bilateral ventriculomegaly on ultrasound, later showed smooth cerebral surface and cortical thickening on MRI, suggesting fetal LIS.

Postnatal phenotypic analysis confirmed that LIS is primarily characterized by severe neurological impairment, with a high incidence of epilepsy, GDD and ID. Moreover, the co-occurrence of abnormalities in the head and neck, skeletal and genitourinary systems indicates that LIS may be part of broader syndromic conditions involving multisystem developmental defects. Therefore, in cases with a prenatal diagnosis or strong suspicion of LIS, early neurodevelopmental monitoring and timely interventions are essential. A multidisciplinary follow-up system should also be established to manage associated anomalies and improve postnatal outcomes.

This study significantly advances our understanding of the genetic landscape and phenotypic correlations of LIS, yet several important unanswered questions remain. Future research could further investigate the mechanisms behind the novel associations identified in this study, such as *NPRL3* and *DARS2* genes, to elucidate their roles in LIS pathogenesis. Additionally, while WES demonstrated high diagnostic yield, it remains unclear how the clinical outcomes correlate with specific genetic variants across larger cohorts. Future studies should focus on validating these genetic findings in diverse populations and exploring genotype−phenotype correlations with a particular focus on rare variants.

A key strength of this study is its comprehensive integration of prenatal and postnatal phenotypic data with genetic profiling, offering a deeper understanding of LIS’s genetic heterogeneity and phenotypic variability. The study’s systematic literature review and comparative analysis further enhance the validity of its findings.

However, the study also has several limitations. First, as a retrospective analysis, it is subject to recall and selection biases. Additionally, among the 80 reviewed studies, LIS subtype classification based on imaging was often inconsistent or absent, so our analysis focused on the broader LIS phenotypic spectrum rather than specific subtypes. Additionally, most fetuses diagnosed prenatally with LIS or suspected LIS underwent pregnancy termination, leading to limited postnatal phenotype data, which may have biased the phenotype spectrum toward surviving cases.

## Conclusions

This study highlights the significant genetic heterogeneity, phenotypic complexity and diagnostic challenges of LIS by integrating data from our cohort and the published literature. We developed a comprehensive genetic aetiology classification framework for LIS and identified novel associations with non-canonical genes such as *NPRL3* and *DARS2*. With a high diagnostic yield of 79.04%, we recommend WES as the first-line genetic test. Furthermore, the establishment of an integrated prenatal imaging-molecular diagnostic system, along with a postnatal multidisciplinary model, is crucial for improving prognosis assessment, clinical decision-making, and genetic counselling.

## Supplementary Material

fcag069_Supplementary_Data

## Data Availability

The data that support the findings of this study are not publicly available as the information contained could compromise the privacy of research participants. Further inquiries can be directed to the corresponding author.
