# Supplementary material for: Genetic landscape and phenotypic correlations of lissencephaly: prenatal and postnatal insights
Source: Brain Commun. 2026 Mar 6;8(2):fcag069. doi: 10.1093/braincomms/fcag069 (PMC12993814; doi:10.1093/braincomms/fcag069)
Supplement: fcag069_Supplementary_Data [file fcag069_supplementary_data.zip › Supplementary Figure 1 PRISMA flow diagram..docx]

**Supplementary Figure 1** PRISMA flow diagram. Abbreviations: ACMG, American College of Medical Genetics and Genomics; LIS, lissencephaly; CMA, chromosomal microarray analysis; NGS, next-generation sequencing
