# Supplementary material for: Genetic landscape and phenotypic correlations of lissencephaly: prenatal and postnatal insights
Source: Brain Commun. 2026 Mar 6;8(2):fcag069. doi: 10.1093/braincomms/fcag069 (PMC12993814; doi:10.1093/braincomms/fcag069)
Supplement: fcag069_Supplementary_Data [file fcag069_supplementary_data.zip › Supplementary File 1 Detailed process of one-stop prenatal diagnosis..docx]

**Supplementary File 1** Detailed process of one-stop prenatal diagnosis.

**1.1 Sample preparation**

Depending on gestational age, fetal samples were collected from amniocytes, or cord blood, with parental and relative samples from peripheral blood. Genomic DNA extraction used the Qiagen DNA Blood Midi/Mini kit (Qiagen GmbH, Hilden, Germany) per manufacturer's instructions, following informed consent.

**1.2 Testing for chromosomal disorders and copy number variants**

Following informed consent, fetal samples underwent quantitative fluorescent polymerase chain reaction (QF-PCR) to exclude maternal cell contamination and rapidly detect aneuploidies (chromosomes 13, 18, 21, X, Y). chromosomal microarray analysis (CMA) utilized Affymetrix CytoScan HD/750K arrays (Affymetrix, Santa Clara, CA, USA), featuring single nucleotide polymorphism (SNP) array and array-based comparative genomic hybridization (aCGH) platforms at 10 and 100 kb resolutions, respectively, aligned to GRCh37/hg19 reference genome. The process has been described in detail elsewhere^1^.

**1.3 Testing for single gene disorders**

After obtaining written informed consent, we performed trio exome sequencing (trio-ES) on the fetus and parental samples. First, we conducted targeted enrichment of DNA samples using the Agilent SureSelect human exome capture probes (V6, Life Technologies, Carlsbad, CA, USA) as per the manufacturer's protocol. The DNA library was subsequently sequenced using Illumina HiSeq2500, HiSeq Xten, or NovaSeq platforms, generating 150 bp paired-end reads.

**1.4 Detailed process of analyzing and interpreting ES data**

After informed consent, genomic DNA was extracted from amniocytes, and cord blood using the Qiagen DNA Blood Midi/Mini kit (Qiagen GmbH, Hilden, Germany) according to the manufacturer’s protocol. Blood samples from the parents were obtained concurrently. Targeted enrichment of the DNA sample was conducted using Agilent SureSelect human exome capture probes (V6, Life Technologies, Carlsbad, CA, USA) according to the manufacturer's protocol. The DNA library was sequenced on a HiSeq XTen or Illumina Novaseq 6000 system (Illumina, Inc.) to obtain 150 bp paired‐end reads.

Raw FASTQ reads were filtered by using Trimmomatic^2^ (v0.36) or fastp^3^ (v0.20/v0.23) to remove low quality and adapter contaminated reads, leaving clean reads aligned to the human reference genome (GRCh37/hg19) with BWA^4^ (v0.7.17) mem algorithm, with Samtools^5^ (v1.3.1/v1.9) and Picard (v2.17.1) converted to BAM format and PCR duplicates were discarded. Genome Analysis Toolkit^6^ (GATK v3.6/v3.8) was employed for local indel realignment, base quality recalibration and HaplotypeCaller variant calling. Variant annotation was conducted with Ensembl’s Variant Effect Predictor^7^ (VEP v85/v104) and Annovar^8^ (v2017Jul17/v2020Apr01). Allele frequency information from 1000 Genome Project (1000G Phase 3 v5a), Genome Aggregation Database (gnomAD r2.1/v2.1.1), Exome Aggregation Consortium (ExAC r0.3.1) and the Exome Sequencing Project (ESP v2) were annotated. Multiple software such as SIFT^9^, Polyphen2^10^, MutationTaster^11^, MutationAssessor^12^, Provean^13^, CADD^14^ and REVEL^15^ were applied for protein function prediction and Human Splicing Finder^16^, MaxEntScan^17^, NNSplice^18^, GeneSplicer^19^ and SpliceAI^20^ were performed to assess potential impacts on splicing. Gene/variants were additionally annotated according to ClinVar, ClinGen, the professional version of the Human Gene Mutation Database (HGMD professional v2018.2 & v2021.2), previously associated diseases (based on Online Mendelian Inheritance in Man and Orphanet), and known functional domain data (according to UniProtKB and Human Protein Reference Database). Besides, the imprinted gene (Geneimprint and MetaImprint database) and genome segmental duplication region (downloaded from the UCSC genome browser) were added to the VCF file annotation. Two aspects were evaluated for gender determination, average depth of specific genes on chromosome Y and the heterozygous variants percentage on chromosome X. Then KING^21^ and PLINK^22^ were used to confirm the family pedigree relationship.

Quality control for each sample included an average depth of > 60X and > 90% targeted region with at least 20X in this study. Variants with poor quality were discarded if meeting one of the following criteria, (1) with a depth (DP) <5X; (2) alternate allele proportion (AAP) <0.25; (3) mapping quality (MQ) <40; (4) genotype quality (GQ) <50. All the annotated variants, excluding low quality ones were subject to downstream analysis with the in-house script. Variants with a minor allele frequency (MAF) >5% were filtered out except for those in HGMD, ClinVar and ClinGen BA1 exception list^23^ (BA1). Next, we mainly focused on genomic regions known or likely associated with the disease. Based on VEP functional consequence, potential protein-altering variants (e.g., missense, start loss, stop gain/loss, frameshift, in-frame insertion/deletion, or canonical splice-site) were retained. To aid data interpretation, major indications for ES for each fetus were extracted from clinical notes and converted into the standard Human Phenotype Ontology (HPO) terms.

A genotype-driven short rare variant list was prioritized for each trio with the help of local population data (more than 10,000 individuals including both patients and healthy individuals), (1) dominant *de novo* variants; (2) recessive homozygous variants (no homozygotes in the gnomAD2.1 and internal healthy controls); (3) recessive compound heterozygous variants; (4) *De novo* X chromosome variants or rare hemizygous variants inherited from mother; (5) known disease-causing alleles (ClinVar 3- or 4- star variants); (6) predicted truncating variants (nonsense, frameshift, canonical splice sites) with extremely low allele frequency (<0.01%). This short gene/variant list was then reviewed for clinical correlation and potentially relevant variants were classified based on the American College of Medical Genetics and Genomics (ACMG) guideline^24^ and ClinGen VCEP gene-specific criteria (when applicable)^25-32^. In addition, *bona fide* disease-causing variants (unrelated to fetal phenotype) with zygosity consistent with disease mode of inheritance in ACMG SF2.0 and childhood-onset disease genes were categorized as potential secondary or incidental findings.

Next, for unsolved cases without a clear answer to the clinical question, a comprehensive review of all rare variants in genes potentially related to clinical indications for prenatal diagnosis was performed with the aid of HPO matching. A gene was considered associated with the fetal anomalies meeting one of the following conditions, the clinical phenotypes of the disease gene should: (1) match HPO entry of the fetal phenotype; (2) match the superclass based on HPO or clinical synopsis in OMIM database; (3) be reported in previous cases manifesting the same or similar phenotypes of the fetuses.

In both steps, ES results were classified into five tiers: (1) positive diagnostic result: P/LP variants identified in a disease gene that can interpret (partly or fully) the fetal phenotype; (2) inconclusive: variant of unknown significance (VUS) identified in a disease gene which can explain (partly or fully) the fetal phenotype; (3) incidental findings (IFs): P/LP variants identified in childhood-onset disease gene, unrelated to fetal phenotype; (4) secondary findings (SFs): P/LP variants identified in genes unrelated to fetal phenotype, according to ACMG recommended list^33, 34^; (5) candidate genes: variants (primarily *de novo*) predicted to be deleterious and absent in general population, identified in undefined disease genes that have a paralog gene or previously published data to support the association with fetal anomalies, or based on animal model and tissue expression.

The ES report included positive diagnostic and inconclusive results related to primary prenatal indications. Incidental and secondary findings with a childhood-onset disease were also included in the report, based on consensus between laboratory and clinicians. Secondary findings with a late-onset disease were not routinely reported.

**Tools:**

Trimmomatic: http://www.usadellab.org/cms/?page=trimmomatic

Fastp: https://github.com/OpenGene/fastp

BWA: https://bio-bwa.sourceforge.net/

Samtools: https://samtools.sourceforge.net/

Picard: https://broadinstitute.github.io/picard/

Genome Analysis Toolkit: https://gatk.broadinstitute.org/hc/en-us

Ensembl’s Variant Effect Predictor: https://www.ensembl.org/info/docs/tools/vep/index.html

Annovar: https://annovar.openbioinformatics.org/en/latest/

SIFT: https://sift.bii.a-star.edu.sg/

Polyphen2: http://genetics.bwh.harvard.edu/pph2/

MutationTaster: https://www.mutationtaster.org/

MutationAssessor: http://mutationassessor.org/r3/

Provean: https://www.jcvi.org/research/provean

CADD: https://cadd.gs.washington.edu/

REVEL: https://sites.google.com/site/revelgenomics/

Human Splicing Finder: http://www.umd.be/HSF3/HSF.shtml

MaxEntScan: http://hollywood.mit.edu/burgelab/maxent/Xmaxentscan_scoreseq.html

NNSplice: http://www.fruitfly.org/seq_tools/splice.html

GeneSplicer: https://ccb.jhu.edu/software/genesplicer/

SpliceAI: https://github.com/Illumina/SpliceAI

KING: https://www.kingrelatedness.com/manual.shtml

PLINK: https://www.cog-genomics.org/plink/

**References**

1. Huang R, Fu F, Guo F, et al. Prenatal diagnosis of polycystic renal diseases: diagnostic yield, novel disease-causing variants, and genotype-phenotype correlations. *Am J Obstet Gynecol MFM*. Jan 2024;6(1):101228. doi:10.1016/j.ajogmf.2023.101228

2. Bolger AM, Lohse M, Usadel B. Trimmomatic: a flexible trimmer for Illumina sequence data. *Bioinformatics*. Aug 1 2014;30(15):2114-20. doi:10.1093/bioinformatics/btu170

3. Chen S, Zhou Y, Chen Y, Gu J. fastp: an ultra-fast all-in-one FASTQ preprocessor. *Bioinformatics*. Sep 1 2018;34(17):i884-i890. doi:10.1093/bioinformatics/bty560

4. Li H, Durbin R. Fast and accurate short read alignment with Burrows-Wheeler transform. *Bioinformatics*. Jul 15 2009;25(14):1754-60. doi:10.1093/bioinformatics/btp324

5. Li H, Handsaker B, Wysoker A, et al. The Sequence Alignment/Map format and SAMtools. *Bioinformatics*. Aug 15 2009;25(16):2078-9. doi:10.1093/bioinformatics/btp352

6. McKenna A, Hanna M, Banks E, et al. The Genome Analysis Toolkit: a MapReduce framework for analyzing next-generation DNA sequencing data. *Genome Res*. Sep 2010;20(9):1297-303. doi:10.1101/gr.107524.110

7. McLaren W, Gil L, Hunt SE, et al. The Ensembl Variant Effect Predictor. *Genome Biol*. Jun 6 2016;17(1):122. doi:10.1186/s13059-016-0974-4

8. Wang K, Li M, Hakonarson H. ANNOVAR: functional annotation of genetic variants from high-throughput sequencing data. *Nucleic Acids Res*. Sep 2010;38(16):e164. doi:10.1093/nar/gkq603

9. Sim NL, Kumar P, Hu J, Henikoff S, Schneider G, Ng PC. SIFT web server: predicting effects of amino acid substitutions on proteins. *Nucleic Acids Res*. Jul 2012;40(Web Server issue):W452-7. doi:10.1093/nar/gks539

10. Adzhubei IA, Schmidt S, Peshkin L, et al. A method and server for predicting damaging missense mutations. *Nat Methods*. Apr 2010;7(4):248-9. doi:10.1038/nmeth0410-248

11. Schwarz JM, Cooper DN, Schuelke M, Seelow D. MutationTaster2: mutation prediction for the deep-sequencing age. *Nat Methods*. Apr 2014;11(4):361-2. doi:10.1038/nmeth.2890

12. Reva B, Antipin Y, Sander C. Predicting the functional impact of protein mutations: application to cancer genomics. *Nucleic Acids Res*. Sep 1 2011;39(17):e118. doi:10.1093/nar/gkr407

13. Choi Y, Chan AP. PROVEAN web server: a tool to predict the functional effect of amino acid substitutions and indels. *Bioinformatics*. Aug 15 2015;31(16):2745-7. doi:10.1093/bioinformatics/btv195

14. Kircher M, Witten DM, Jain P, O'Roak BJ, Cooper GM, Shendure J. A general framework for estimating the relative pathogenicity of human genetic variants. *Nat Genet*. Mar 2014;46(3):310-5. doi:10.1038/ng.2892

15. Ioannidis NM, Rothstein JH, Pejaver V, et al. REVEL: An Ensemble Method for Predicting the Pathogenicity of Rare Missense Variants. *Am J Hum Genet*. Oct 6 2016;99(4):877-885. doi:10.1016/j.ajhg.2016.08.016

16. Desmet FO, Hamroun D, Lalande M, Collod-Beroud G, Claustres M, Beroud C. Human Splicing Finder: an online bioinformatics tool to predict splicing signals. *Nucleic Acids Res*. May 2009;37(9):e67. doi:10.1093/nar/gkp215

17. Yeo G, Burge CB. Maximum entropy modeling of short sequence motifs with applications to RNA splicing signals. *J Comput Biol*. 2004;11(2-3):377-94. doi:10.1089/1066527041410418

18. Reese MG, Eeckman FH, Kulp D, Haussler D. Improved splice site detection in Genie. *J Comput Biol*. Fall 1997;4(3):311-23. doi:10.1089/cmb.1997.4.311

19. Pertea M, Lin X, Salzberg SL. GeneSplicer: a new computational method for splice site prediction. *Nucleic Acids Res*. Mar 1 2001;29(5):1185-90. doi:10.1093/nar/29.5.1185

20. Jaganathan K, Kyriazopoulou Panagiotopoulou S, McRae JF, et al. Predicting Splicing from Primary Sequence with Deep Learning. *Cell*. Jan 24 2019;176(3):535-548 e24. doi:10.1016/j.cell.2018.12.015

21. Manichaikul A, Mychaleckyj JC, Rich SS, Daly K, Sale M, Chen WM. Robust relationship inference in genome-wide association studies. *Bioinformatics*. Nov 15 2010;26(22):2867-73. doi:10.1093/bioinformatics/btq559

22. Chang CC, Chow CC, Tellier LC, Vattikuti S, Purcell SM, Lee JJ. Second-generation PLINK: rising to the challenge of larger and richer datasets. *Gigascience*. 2015;4:7. doi:10.1186/s13742-015-0047-8

23. Ghosh R, Harrison SM, Rehm HL, Plon SE, Biesecker LG, ClinGen Sequence Variant Interpretation Working G. Updated recommendation for the benign stand-alone ACMG/AMP criterion. *Hum Mutat*. Nov 2018;39(11):1525-1530. doi:10.1002/humu.23642

24. Richards S, Aziz N, Bale S, et al. Standards and guidelines for the interpretation of sequence variants: a joint consensus recommendation of the American College of Medical Genetics and Genomics and the Association for Molecular Pathology. *Genet Med*. May 2015;17(5):405-24. doi:10.1038/gim.2015.30

25. Kelly MA, Caleshu C, Morales A, et al. Adaptation and validation of the ACMG/AMP variant classification framework for MYH7-associated inherited cardiomyopathies: recommendations by ClinGen's Inherited Cardiomyopathy Expert Panel. *Genet Med*. Mar 2018;20(3):351-359. doi:10.1038/gim.2017.218

26. Gelb BD, Cave H, Dillon MW, et al. ClinGen's RASopathy Expert Panel consensus methods for variant interpretation. *Genet Med*. Nov 2018;20(11):1334-1345. doi:10.1038/gim.2018.3

27. Shen J, Oza AM, Del Castillo I, et al. Consensus interpretation of the p.Met34Thr and p.Val37Ile variants in GJB2 by the ClinGen Hearing Loss Expert Panel. *Genet Med*. Nov 2019;21(11):2442-2452. doi:10.1038/s41436-019-0535-9

28. Oza AM, DiStefano MT, Hemphill SE, et al. Expert specification of the ACMG/AMP variant interpretation guidelines for genetic hearing loss. *Hum Mutat*. Nov 2018;39(11):1593-1613. doi:10.1002/humu.23630

29. Mester JL, Ghosh R, Pesaran T, et al. Gene-specific criteria for PTEN variant curation: Recommendations from the ClinGen PTEN Expert Panel. *Hum Mutat*. Nov 2018;39(11):1581-1592. doi:10.1002/humu.23636

30. Abou Tayoun AN, Pesaran T, DiStefano MT, et al. Recommendations for interpreting the loss of function PVS1 ACMG/AMP variant criterion. *Hum Mutat*. Nov 2018;39(11):1517-1524. doi:10.1002/humu.23626

31. Lee K, Krempely K, Roberts ME, et al. Specifications of the ACMG/AMP variant curation guidelines for the analysis of germline CDH1 sequence variants. *Hum Mutat*. Nov 2018;39(11):1553-1568. doi:10.1002/humu.23650

32. Zastrow DB, Baudet H, Shen W, et al. Unique aspects of sequence variant interpretation for inborn errors of metabolism (IEM): The ClinGen IEM Working Group and the Phenylalanine Hydroxylase Gene. *Hum Mutat*. Nov 2018;39(11):1569-1580. doi:10.1002/humu.23649

33. Miller DT, Lee K, Chung WK, et al. ACMG SF v3.0 list for reporting of secondary findings in clinical exome and genome sequencing: a policy statement of the American College of Medical Genetics and Genomics (ACMG). *Genet Med*. Aug 2021;23(8):1381-1390. doi:10.1038/s41436-021-01172-3

34. Kalia SS, Adelman K, Bale SJ, et al. Recommendations for reporting of secondary findings in clinical exome and genome sequencing, 2016 update (ACMG SF v2.0): a policy statement of the American College of Medical Genetics and Genomics. *Genet Med*. Feb 2017;19(2):249-255. doi:10.1038/gim.2016.190
