# Supplementary material for: Genetic landscape and phenotypic correlations of lissencephaly: prenatal and postnatal insights
Source: Brain Commun. 2026 Mar 6;8(2):fcag069. doi: 10.1093/braincomms/fcag069 (PMC12993814; doi:10.1093/braincomms/fcag069)
Supplement: fcag069_Supplementary_Data [file fcag069_supplementary_data.zip › Supplementary Table 1.docx]

**Supplementary Table 1 Prenatal genetic profiling of LIS cases: ES-based variant spectrum and clinical correlations**

| **No.** | **MA (y)** | **GW (wk)** | **Fetal phenotype** | **Gene**  **(OMIM)** | **Reference sequence** | **Chromosomal Loci (GRCh37/hg19)** | **Variant** | **Zygosity; Origin; Inheritance** | **ACMG classification** | **Disorder (OMIM)** | **Outcome** |
| --- | --- | --- | --- | --- | --- | --- | --- | --- | --- | --- | --- |
| 7 | 26 | 34.4 | Pachygyria; Ventriculomegaly (B); Polyhydramnios | *PAFAH1B1* (601545) | NM_000430.4 | 17:2570469 | c.377del (p.Ala126ValfsTer47) | Het; De novo; AD | P (PVS1, PS2, PM2_Supporting) | LIS1(607432) | TOP |
| 8 | 33 | 29.0 | Agyria | *FLNA* (300017) | NM_001110556.2 | X:153590129 | c.2853T>G (p.Tyr951Ter) | Het; De novo; XLR/XLD/XL | P (PVS1, PS2_Supporting, PM2_Supporting) | FGS2 (300321); CVDPX (314400); CIIPX (300048); FMD1 (305620); PVNH1 (300049); MNS (309350); OPD1 (311300); OPD2 (304120); TOD (300244) | TOP |
| 9 | 20 | 28.0 | Agyria; Hydrocephalus (B); ACC; Microcephaly; TE (B) | *PDHA1* (300502) | NM_000284.4 | X:19377058 | c.934_940del (p.Ser312ValfsTer12) | Het; De novo; XLD | P (PVS1_Strong, PS2, PM2_Supporting, PS4_Supporting) | PDHAD (312170) | TOP |
| 10 | 34 | 25.7 | Pachygyria? Arachnoid cyst; Pericardial effusion | *ACTB* (102630) | NM_001101.5 | 7:5568284 | c.430G>A (p.Ala144Thr) | Het; De novo; AD | LP (PS2, PP2, PM2_Supporting, PM1_Supporting, PP3_Moderate) | BRWS1 (243310); BNS (604919); CSMH (620470); DDS1 (607371); THC8 (620475) | TOP |
| 11 | 36 | 24.0 | Agyria; Microcephaly; Hypoplasia of the lungs; Scoliosis; Abnormal posture of the limbs | *DARS2* (610956) | NM_018122.5 | 1:173808532  1:173826767 | c.868C>T (p.Gln290Ter)  c.1862T>C (p.Val621Ala) | Het; Mat; AR  Het; Pat; AR | LP (PVS1, PM2_Supporting)  VUS (PM2_Supporting) | LBSL (611105) | TOP |
| 12 | 31 | 31.3 | Pachygyria? Ventriculomegaly (B) | *HDAC6* (300272) | NM_006044.4 | X:48682196 | c.3303+1G>C | Hemi; Mat; XLD | VUS (PVS1_Moderate, PM2_Supporting) | CPDBHM (300863) | Live Birth |
| 13 | 24 | 25.7 | Pachygyria; Ventriculomegaly (L); Subependymal cysts | *SLC5A6* (604024) | NM_021095.4 | 2:27429747  2:27428274 | c.457A>G (p.Met153Val)  c.678C>T (p.Gly226=) | Het; Pat; AR  Het; Mat; AR | VUS (PM2_Supporting, PP3)  VUS (PM2_Supporting) | COMNB (619903); SMVTD (618973) | TOP |
| 14 | 28 | 29.7 | Pachygyria? Ventriculomegaly (R); Subependymal cysts | *CREBBP (600140)* | NM_004380.3 | 16:3843471 | c.1132C>T (p.Arg378Trp) | Het; Pat; AD | VUS (PM2_Supporting, PP2, PP3) | MKHK1 (618332); RSTS1 (180849) | TOP |
| 15 | 29 | 35.0 | Pachygyria; FGR | *FRMPD4* (300838) | NM_001368397.1 | X:12725613 | c.1313C>T (p.Thr438Ile) | Hemi; Mat; XL | VUS (PM2_Supporting) | XLID104 (300983) | TOP |
| 16 | 22 | 29.0 | Agyria | Negative | | | | | | | TOP |
| 17 | 33 | 34.7 | Agyria; Hydrocephalus (B); Microcephaly | Negative | | | | | | | TOP |
| 18 | 31 | 24.4 | Agyria; Ventriculomegaly (B); VSD | Negative | | | | | | | TOP |
| 19 | 34 | 29.0 | Agyria; Short long bone | Negative | | | | | | | TOP |
| 20 | 30 | 32.0 | Pachygyria; DWS; Ventriculomegaly (B) | Negative | | | | | | | Live Birth |

LIS: Lissencephaly; ES: exome sequencing; MA: Maternal age; GW: Gestational weeks; B: Bilateral; Het: Heterozygous; AD: Autosomal dominant; P: Pathogenic; LIS1: Lissencephaly 1; TOP: Termination of pregnancy; XLR: X-Linked recessive; XLD: X-Linked dominant; XL: X-Linked; FGS2: FG syndrome 2; CVDPX: Cardiac valvular dysplasia, X-linked; CIIPX: Chronic idiopathic intestinal pseudoobstruction, X-linked; FMD1: Frontometaphyseal dysplasia 1; PVNH1: Periventricular nodular heterotopia 1; MNS: Melnick-Needles syndrome; OPD1: Otopalatodigital syndrome type I; OPD2: Otopalatodigital syndrome type II; TOD: Terminal osseous dysplasia; ACC: Agenesis of corpus callosum; TE: Talipes equinovarus; PDHAD: Pyruvate dehydrogenase E1-alpha deficiency; BRWS1: Baraitser-Winter syndrome 1; BNS: Becker nevus syndrome; CSMH: Congenital smooth muscle hamartoma; DDS1: Dystonia-deafness syndrome 1; THC8: Thrombocytopenia 8 with dysmorphic features and developmental delay; Mat: Maternal inherited; AR: Autosomal recessive; LP: Likely pathogenic; Pat: Paternal inherited; VUS: Variants of unknown significance; LBSL: leukoencephalopathy with brainstem and spinal cord involvement and lactate elevation; Hemi: hemizygous; CPDBHM: Chondrodysplasia with platyspondyly, distinctive brachydactyly, hydrocephaly, and microphthalmia; L: left; COMNB: Childhood-onset biotin-responsive peripheral motor neuropathy; SMVTD: Sodium-dependent multivitamin transporter deficiency; R: right; MKHK1: Menke-Hennekam syndrome 1; RSTS1: Rubinstein-Taybi syndrome 1; FGR: Fetal growth restriction; XLID104: X-linked intellectual developmental disorder-104; VSD: Ventricular septal defect; DWS: Dandy-Walker syndrome.
