# Supplementary material for: Genetic landscape and phenotypic correlations of lissencephaly: prenatal and postnatal insights
Source: Brain Commun. 2026 Mar 6;8(2):fcag069. doi: 10.1093/braincomms/fcag069 (PMC12993814; doi:10.1093/braincomms/fcag069)
Supplement: fcag069_Supplementary_Data [file fcag069_supplementary_data.zip › Supplementary Table 2.docx]

**Supplementary Table 2 Postnatal Diagnosis of LIS Cases: genotype-phenotype associations and neurodevelopmental outcomes**

| **No.** | **Age of onset** | **Sex** | **Postnatal main phenotype** | **MRI results** | **Prenatal phenotype** | **Delivery status** | **Gene**  **(OMIM)** | **Reference sequence** | **Chromosomal Locus (GRCh37/hg19)** | **Variant** | **Inheritance** | **ACMG classification** |
| --- | --- | --- | --- | --- | --- | --- | --- | --- | --- | --- | --- | --- |
| 21 | 5 months | Female | GDD, Seizure | Pachygyria | Ventriculomegaly (R); Polyhydramnios | Full-term C-section | *-* | - | 17:2,246,504-2,610,163 | 17p13.3 deletion (0.36Mb) | Deletion, De novo | P |
| 22 | 3 months | Male | GDD, Seizure | Pachygyria | Non-specific | Full-term vaginal delivery | *-* | - | 17:2,323,264-2,541,614 | 17p13.3 deletion (0.22Mb) | Deletion, De novo | P |
| 23 | 6 months | Male | GDD | Pachygyria, CH | Non-specific | Full-term vaginal delivery | *-* | - | 3:195,778,813-197,717,547 | 3q29 duplication (1.94Mb) | Duplication, De novo | LP |
| 24 | 1 month | Male | GDD, Seizure | Pachygyria, ACC | Non-specific | Full-term vaginal delivery | *PAFAH1B1*  (601545) | NM_000430.4 | 17:2570472 | c.379T>C (p.Ser127Pro) | Het, De novo, AD | P (PS2, PP4, PM2_Supporting, PP3_Moderate) |
| 25 | 7 months | Female | GDD, Seizure | Pachygyria | Non-specific | Full-term C-section | *PAFAH1B1*  (601545) | NM_000430.4 | 17:2568673 | c.41_42dup (p.Ile15LeufsTer2) | Het, De novo, AD | P (PVS1, PS2_Moderate, PM2_Supporting) |
| 26 | 1 month | Female | Seizure | Agyria | Non-specific | Full-term vaginal delivery | *PAFAH1B1*  (601545) | NM_000430.4 | 17:2577389 | c.714_722del (p.Met239_Arg241del) | Het, De novo, AD | LP (PS2, PM4, PM2_ Supporting) |
| 27 | 6 months | Female | GDD, Seizure | Pachygyria | Ventriculomegaly (L), SEC (L) | Full-term vaginal delivery | *PAFAH1B1*  (601545) | NM_000430.4 | 17:2568673 | c.41_42dup (p.Ile15LeufsTer2) | Het, De novo, AD | P (PVS1, PS2_Moderate, PM2_Supporting) |
| 28 | 7 months | Female | GDD, Dystonia | Pachygyria | FGR | Full-term C-section, Fetal distress | *PAFAH1B1*  (601545) | NM_000430.4 | 17:2583598 | c.1146dup (p.Val383CysfsTer46) | Het, De novo, AD | LP (PS2, PVS1_Moderate, PM2_Supporting) |
| 29 | 18 days | Male | Seizure | Pachygyria, WMI | 36 weeks: Decreased fetal movement | Full-term C-section, Fetal distress | *NPRL3*  (600928) | NM_001077350.3 | 16:169124 | c.318+1G>T | Het, De novo, AD | P (PVS1, PS2_Moderate, PM2_Supporting) |
| 30 | 4 days | Male | Dysphagia, Seizure | Pachygyria | FGR, Oligohydramnios | Full-term C-section, Fetal distress | *OSGEP*  (610107) | NM_017807.4 | 14:20916116 | c.740G>A (p.Arg247Gln) | Homo, Mat+Pat, AR | P (PM3_VeryStrong, PS3_Supporting, PP3, PM2_Supporting PM1_Supporting, PP1) |
| 31 | 6 months | Male | GDD, Seizure | Pachygyria | Non-specific | Full-term vaginal delivery | *TUBB2A*  (615101) | NM_001069.3 | 6:3154707 | c.728C>T (p.Pro243Leu) | Het, De novo, AD | LP (PM1, PP2, PM2_Supporting, PP3_Moderate) |
| 32 | 7 months | Female | GDD, Seizure, Anal atresia | Pachygyria, CH | Non-specific | Full-term C-section | *RELN*  (600514) | NM_005045.4 | 7:103252074 | c.2879G>A (p.Trp960Ter) | Homo, Mat+Pat, AR | LP (PVS1, PM2_Supporting) |
| 33 | 3 months | Female | GDD, Seizure | Pachygyria | Non-specific | Full-term vaginal delivery | *ADGRG1*  (604110) | NM_201525.4 | 16:57685259  16:57694734 | c.215del (p.Pro72LeufsTer41)  c.1610G>A (p.Gly537Asp) | Het, Mat, AR  Het, Pat, AR | LP (PVS1, PM2_Supporting)  VUS (PM2_Supporting) |
| 34 | 5 days | Male | Seizure | Pachygyria, ACC | Non-specific | Full-term C-section, Placental abruption | *NPRL2*  (607072) | NM_006545.5 | 3:50388048-50388050 | c.34_36del (p.Phe12del) | Het, Pat, AD | VUS (PM4, PM2_Supporting) |
| 35 | 1 year | Male | Seizure, Infantile spasms | Pachygyria | Ventriculomegaly (B) | Full-term vaginal delivery | Negative | | | | | |
| 36 | 1 month | Female | GDD, Microcephaly | Pachygyria, PMG, schizencephaly | Foot eversion (L) | Full-term C-section | Negative | | | | | |
| 37 | 15 hours | Male | Seizures, Intracranial hemorrhage | Pachygyria, Meningitis, Hydrocephalus | Oligohydramnios, Intrauterine infection? | Full-term C-section, Intrapartum fever | Negative | | | | | |
| 38 | at birth | Male | GNH, Seizures, Cholestasis | Pachygyria, PMG, SEC | Ventriculomegaly (L), SEC (L) | Full-term C-section, Neonatal asphyxia | Negative | | | | | |
| 39 | 1 month | Female | GDD, Microcephaly, Micrognathia, Foot eversion (L) | Pachygyria | FGR | Full-term vaginal delivery, low birth weight | Negative | | | | | |
| 40 | 3 months | Male | GDD, Seizure | Pachygyria | Non-specific | Full-term vaginal delivery | Negative | | | | | |

LIS: Lissencephaly; F: Female; GDD: Global developmental delay (HP:0001263); R: Right; P: Pathogenic; M: Male; CH: Cerebellar hypoplasia; ACC: Agenesis of the corpus callosum; Het: Heterozygous; AD: Autosomal dominant; C-section: Cesarean section; L: Left; SEC: Subependymal cyst; FGR: Fetal growth restriction; LP: Likely pathogenic; WMI: White matter injury; homo: Homozygote; Mat: Maternal inherited; Pat: Paternal inherited; AR: Autosomal recessive; VUS: Variants of unknown significance; B: Bilateral; GNH: Generalized neonatal hypotonia (HP:0008935); PMG: Polymicrogyria.
