# Supplementary material for: Genetic landscape and phenotypic correlations of lissencephaly: prenatal and postnatal insights
Source: Brain Commun. 2026 Mar 6;8(2):fcag069. doi: 10.1093/braincomms/fcag069 (PMC12993814; doi:10.1093/braincomms/fcag069)
Supplement: fcag069_Supplementary_Data [file fcag069_supplementary_data.zip › Supplementary_File_2_Detailed_process_of_literature_search_and_review..docx]

**Supplementary File 2** Detailed process of literature search and review.

**2.1 Study Selection and Data Extraction**

A systematic search was conducted in PubMed, Scopus, and Web of Science up to April 7, 2025, to identify studies on LIS, CMA, or next-generation sequencing (NGS). The search used the following Boolean operators: ((Agyria OR Pachygyria OR Subcortical band heterotopia OR Lissencephaly) AND ((Exome sequencing OR Whole exome sequencing) OR (Genome sequencing OR Whole genome sequencing) OR (Chromosomal microarray analysis OR CMA) OR (Copy number variant OR CNV OR Copy number variation) OR (Single nucleotide variant OR SNV OR Point mutation))).

Based on pre-established inclusion and exclusion criteria, two independent reviewers (R.H. and N.Z.) completed the initial screening, blinded to each other. The screening process is as follows: (1) First, the titles and abstracts were reviewed, and studies not meeting the criteria were excluded; (2) Subsequently, the full texts of the selected studies were reviewed to finalize the list of included studies. Any disagreements during the screening process were resolved through discussions with a third reviewer (F.F.) until consensus was reached. The inclusion criteria for studies were: (1) The subjects were fetuses or children diagnosed with LIS in prenatal or postnatal settings; (2) The study used CMA or next-generation sequencing technologies (panel sequencing, exome sequencing, genome sequencing); (3) Clear sequencing data were provided, including identified genes, transcripts, mutation sites, amino acid changes, and ACMG classification. In addition, we excluded review articles, conference abstracts, guidelines, editorials, comments, and opinion articles. However, due to the rarity of LIS and the fact that most existing reports are case studies, case studies were included in this study’s screening. Full-text retrieval was performed for all studies that met the inclusion criteria, and eligibility was assessed using a predefined data extraction form. To prevent omissions, references from the included studies were also reviewed, and no missing literature was found. The study selection process is detailed in Supplementary Figure 1. For studies meeting the inclusion criteria, after full-text review, data extraction was performed using a predefined Excel sheet. The extracted data included the first author, publication year, sequencing method, total number of patients included, number of positive cases, gene locus information, prenatal/postnatal phenotypes, imaging findings, etc. All extracted data are provided in Supplementary File 3.

**2.2 Standardized Annotation Process of Genetic Variants**

To ensure the standardization and data comparability of genetic variant annotations across studies, this research established a systematic integrated analysis workflow. The specific steps are as follows:

1. Systematic Integration of Variants from Literature Sources: Through the literature search and review described above, all genetic variant sites in the included cases were comprehensively collected. In this process, we recorded the transcript versions and genome coordinates used in the original studies and created a temporary database for the original variant sites.
2. Standardization of Genome Versions and Transcript Updates: To eliminate inconsistencies in variant positions caused by different transcript versions, this study reannotated each genetic variant site based on the GRCh37/hg19 reference genome and its related transcript annotation information, ensuring consistency between the transcript version and the reference genome version.
3. Normalization of Human Genome Variation Society (HGVS) Nomenclature: During the reannotation of variants, we strictly followed the HGVS standards to ensure the standardized representation of variant sites. This process includes the following key points: ① Nucleotide Variants: By aligning with the reference genome, we accurately mapped the positions of nucleotide variants, ensuring that all variants followed the HGVS standard format and clearly indicated the type of variant (e.g., missense, nonsense, etc.). ② Amino Acid Variants: For variants in protein-coding regions, we used the latest transcript information to express amino acid changes in HGVS standard format, clearly indicating the amino acid position and substitution type for each variant. ③ Chromosomal Position: By comparing with the latest genome annotation data, we ensured that the chromosomal positions of the variant sites matched the latest reference genome, avoiding positional deviations caused by transcript updates.
4. Construction of a Standardized Variant Database: After completing the annotation process, we constructed a structured variant database containing standardized fields (such as gene symbols, HGVS cDNA/protein nomenclature, GRCh37 chromosomal coordinates, clinical significance classification, etc.). This database provides a reliable data foundation for subsequent analyses (such as variant spectrum heatmaps, genotype-phenotype association analysis, etc.).

**2.3 HPO Standardized Annotation of Clinical Phenotypes**

To eliminate heterogeneity in phenotype descriptions across different studies, this research used the Human Phenotype Ontology (HPO) to annotate and standardize the clinical phenotypes reported in the literature. The specific process is as follows:

1. Collection and Organization of Clinical Phenotypes: Through the literature search and review mentioned above, all case information from the included studies was comprehensively collected, and all relevant clinical phenotype descriptions were extracted and recorded. We specifically focused on differences in phenotype descriptions across studies to ensure that all clinical features of the cases were fully collected and unified.
2. Mapping and Standardization of HPO Terms: Based on the collected clinical phenotype descriptions, the official HPO tool was used to map each phenotype to the most precise HPO term. All phenotype descriptions were standardized according to the definitions provided by HPO, ensuring consistency in phenotype expression. If the phenotype description used in the literature did not directly match an HPO term, we provided appropriate annotations based on the clinical presentation of the phenotype and the definition of the HPO term, ensuring that each clinical phenotype was accurately represented by an HPO term.
3. Selection and Application of HPO Term Versions: This study used the latest version of the HPO database for standard terms, ensuring that all mapped phenotype terms were up-to-date and validated. The use of this version guarantees the timeliness and accuracy of the phenotype data, avoiding inconsistencies caused by version updates.
4. Construction of a Standardized Phenotype Database: Finally, all the mapped HPO terms were integrated into a structured database, ensuring that the clinical phenotype of each case was expressed using a unified HPO term. This database provides standardized data support for subsequent genotype-phenotype association analysis and other related studies.
